# Influence of data sampling methods on the representation of neural spiking activity *in vivo*

**DOI:** 10.1016/j.isci.2022.105429

**Published:** 2022-10-22

**Authors:** Meike E. van der Heijden, Amanda M. Brown, Roy V. Sillitoe

**Affiliations:** 1 Department of Pathology and Immunology, Baylor College of Medicine, Houston, TX, USA; 2 Department of Neuroscience, Baylor College of Medicine, Houston, TX, USA; 3 Department of Pediatrics, Baylor College of Medicine, Houston, TX, USA; 4 Development, Disease Models and Therapeutics Graduate Program, Baylor College of Medicine, Houston, TX, USA; 5 Jan and Dan Duncan Neurological Research Institute at Texas Children’s Hospital, Houston, TX, USA

**Keywords:** Biological sciences, Biological sciences research methodologies, Methodology in biological sciences, Neuroscience

## Abstract

*In vivo* single-unit recordings distinguish the basal spiking properties of neurons in different experimental settings and disease states. Here, we examined over 300 spike trains recorded from Purkinje cells and cerebellar nuclei neurons to test whether data sampling approaches influence the extraction of rich descriptors of firing properties. Our analyses included neurons recorded in awake and anesthetized control mice, and disease models of ataxia, dystonia, and tremor. We find that recording duration circumscribes overall representations of firing rate and pattern. Notably, shorter recording durations skew estimates for global firing rate variability toward lower values. We also find that only some populations of neurons in the same mouse are more similar to each other than to neurons recorded in different mice. These data reveal that recording duration and approach are primary considerations when interpreting task-independent single neuron firing properties. If not accounted for, group differences may be concealed or exaggerated.

## Highlights

• Sampling methods for transient *in vivo* neural recordings affect data representation
• Recording length skews results for neurons with diverse firing rates and patterns
• Short sample durations systematically underestimate overall firing rate variability
• Nested data can mask population variability in some neural subtypes and experiments

## Graphical abstract

**Figure:**
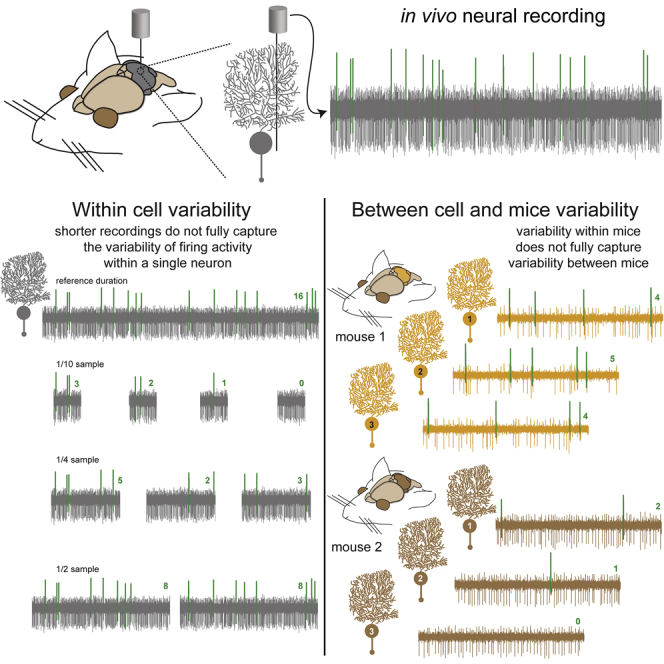

## Introduction

*In vivo* recordings of single neurons are used to examine and compare spiking activity between distinct cell types,^1^^,^^2^^,^^3^ brain regions,^4^ developmental timepoints,^5^^,^^6^ disease models,^7^^,^^8^ across species,^9^ and among human patients.^10^^,^^11^^,^^12^ Many experimental settings render chronic or long-term recordings unfeasible. Experimental constraints, such as when the brain is actively growing, may prevent the implantation of a chronic recording setup.^5^^,^^6^ Therefore, transient recordings may be the best or one’s only chance to record specific cell populations, such as opportune recordings of neurons that are obtained in human patients when electrodes are implanted during deep brain stimulation surgery.^10^^,^^11^^,^^12^ These shorter term recordings often measure task-independent, instantaneous, or basal firing activity. Although such recordings have provided fundamental insight into neural function in health and disease, it remains unclear whether short recordings are rich enough to represent the population, and if they are, what are the specific limitations compared to longer recordings. Even in the absence of using chronic recordings or task-specific responsiveness approaches, many experimenters set a predetermined recording duration or spike number as inclusion criterion for analyses of neural firing properties.^4^^,^^6^^,^^12^^,^^13^ Due to the opportunistic nature of these recordings, experimenters must also decide how many neurons and patients, or animals, to attempt to include in these analyses. Here, we investigate how data sampling strategies may influence the representation of firing properties of single neurons. We analyzed more than 300 experimentally obtained spike trains from two primary populations of cerebellar neurons, Purkinje cells and cerebellar nuclei neurons, which were recorded in control mice as well as in different disease models and behavioral states.

It is necessary to study cell types that are representative of the wide range of baseline firing properties, variety of inputs and outputs, complexity of organization and connectivity, and relevance to disease pathophysiology found in the central nervous system (CNS) in order to determine generalizable impacts of data sampling strategies. The cerebellum has been used to model the development,^14^ evolution,^15^ pathogenesis,^16^ and electrophysiology^17^ of the nervous system at large. The canonical cerebellar circuit is well defined and has stereotyped connectivity between specific neuronal populations,^18^^,^^19^ allowing for the examination of the electrophysiology of identifiable cell populations with known integration into the circuit. The cerebellum has at least two key principal neuron types responsible for information integration, Purkinje cells and nuclei neurons. Purkinje cells integrate incoming motor and sensory information to the cerebellum and form the sole output of the cerebellar cortex. Cerebellar nuclei projection neurons receive major inhibitory input from Purkinje cells and, in turn, form the primary connection between the cerebellum and dozens of other brain regions. These cell types are capable of producing spike activity analogous to what is found elsewhere in the CNS, where spike frequencies can range from hundreds of hertz^20^ to less than one spike per second^21^ and spike patterns can range from tonic, regular firing to irregular or burst firing.^22^ In the cerebellum, both the Purkinje cells and the subtypes of cerebellar nuclei neurons most likely encountered during *in vivo* electrophysiology can fire action potentials at the upper range of what is encountered in the cerebral cortex, ranging from under 30 to over 100 spike/s.^2^^,^^23^ In addition, Purkinje cells also fire a second type of action potential, called the complex spike, which occurs at about 1 spike/s and therefore represents the lower range of frequencies found in the cerebral cortex.^2^^,^^24^ The range of action potential firing patterns is therefore represented across cerebellar cell types. Purkinje cells and nuclei neurons have intrinsic properties that allow them to generate remarkably regular action potentials even without input from other neurons.^25^^,^^26^^,^^27^ However, sensory and motor afferents modify these intrinsic firing properties in the intact cerebellum of live animals,^28^^,^^29^^,^^30^^,^^31^^,^^32^^,^^33^^,^^34^^,^^35^ resulting in highly dynamic firing properties in the *in vivo* circuit.

The extent of the diversity of cell populations in the CNS is still actively being uncovered as demonstrated in recent research^36^ and, therefore, the study of data sampling strategies also requires the use of cell populations with some known degree of heterogeneity. The modular organization of cerebellar circuits provides a known complexity within the firing activity of neurons.^37^^,^^38^^,^^39^ Purkinje cell firing rate and pattern are distinct between at least two populations,^1^^,^^2^^,^^40^^,^^41^ which are consistent with zebrinII molecular expression and specific pre- and post-synaptic partners.^42^^,^^43^^,^^44^ The modular organization further influences the firing activity of the cerebellar nuclei projection neurons,^45^ which are classified as excitatory and inhibitory neurons, each with their specific synaptic partners^46^ and electrophysiological properties.^3^^,^^47^^,^^48^ It is difficult to distinguish between the subtypes of Purkinje cells and nuclei neurons during standard *in vivo* recordings in the cerebellum because the different populations only partially follow anatomical boundaries within the cerebellar cortex and nuclei. Additionally, even though the mean firing rate and pattern are different, the range of these parameters overlaps between molecularly distinct subtypes of Purkinje cells and nuclei neurons.^3^^,^^45^^,^^49^ Therefore, the diversity of firing properties within whole populations of Purkinje cells and nuclei neurons represents intercellular variability, provided by the heterogeneity of intrinsic properties and synaptic inputs, plus intracellular variability, provided by the dynamic impact of the many sensorimotor signals. Thus, the complexity and dynamics of cerebellar function offer an inroad to explore data sampling in both diverse and uniform contexts.

Ultimately, changes in the spiking activity of cerebellar neurons have a direct relevance to neurological disorders. In mouse models of cerebellar movement disorders, the source of cerebellar dysfunction is consistent with changes in the firing rate and/or pattern of Purkinje cell and cerebellar nuclei neuron firing.^7^^,^^13^^,^^50^^,^^51^^,^^52^^,^^53^^,^^54^^,^^55^^,^^56^^,^^57^^,^^58^^,^^59^ Altered basal firing properties have also been reported in the thalamus,^11^ basal ganglia,^60^ and cerebral cortex^61^ during abnormal movements, and during seizures.^62^^,^^63^ The task of recording firing activity from neurons in the awake condition has many challenges and limitations, particularly in movement disorders, as the awake animal may frequently perform involuntary actions that can destabilize neural recordings.^64^ Many experiments make use of anesthetized preparations to address these limitations.^38^ However, the presence of the chosen anesthetic and particular anesthesia regime can also affect the firing features of neurons^65^^,^^66^ and anesthesia-induced immobility could limit sensorimotor-related changes in neural firing.^29^^,^^30^^,^^56^^,^^67^^,^^68^^,^^69^ Likewise, fluctuations in firing properties associated with the relative arousal state of the animal may be relevant to ongoing behavior.^70^^,^^71^^,^^72^ This raises the question, within the constraints of standard experimental settings, how can we ensure that biologically relevant differences are represented in the spiking activity of single neurons when comparisons are made between experimental groups?

In this study, we investigate how experimental constraints on data sampling, such as recording duration and experimental replicates, affect the analysis of *in vivo* neuronal activity. We use two principal neuron types of the cerebellum to examine a diverse set of neuronal spiking properties. We find that recording duration influences the measurement of variability of firing rate over short timescales. Additionally, we find varying degrees of cell-to-cell variability within neural populations. These findings have implications for determining the recording duration and number of cells per animal that are needed to make meaningful comparisons across experimental groups.

## Results

### Influence of sample duration on firing rate and pattern representation

The cerebellum encodes information using a combination of firing rate coding and millisecond precise spike train activity.^73^ Parameters describing the firing rate and pattern are often used to describe the basal firing properties of cerebellar neurons, and to investigate functional differences between cell types or disease states. Inter-spike intervals help define both the firing rate and firing pattern.^74^ The number of inter-spike intervals sampled over a predetermined duration represents the firing rate (spikes/s) and the inter-spike interval timing variation determines the firing pattern or firing rate irregularity. We defined “global irregularity” as a measure of variability relative to the mean firing rate in the recording, also known as the coefficient of variation (CV). CV effectively captures burst activity patterns, longer firing rate fluctuations, and prolonged pauses between spikes. We defined “local irregularity” as the relative difference in inter-spike intervals between adjacent spikes, also known as CV2.^74^ CV2 captures the variability of spike trains. Firing rate, CV, and CV2 are among the most used parameters describing neural activity because they provide direct insight into cerebellar function. For example, cerebellar neurons recorded in mouse models for movement disorders often have profoundly different firing rates, CV, or CV2 compared to neurons recorded in healthy control mice.^7^^,^^38^^,^^75^ Thus, changes in firing rate, CV, and CV2 have direct functional importance and should be captured both accurately and precisely.

**Figure 1:**
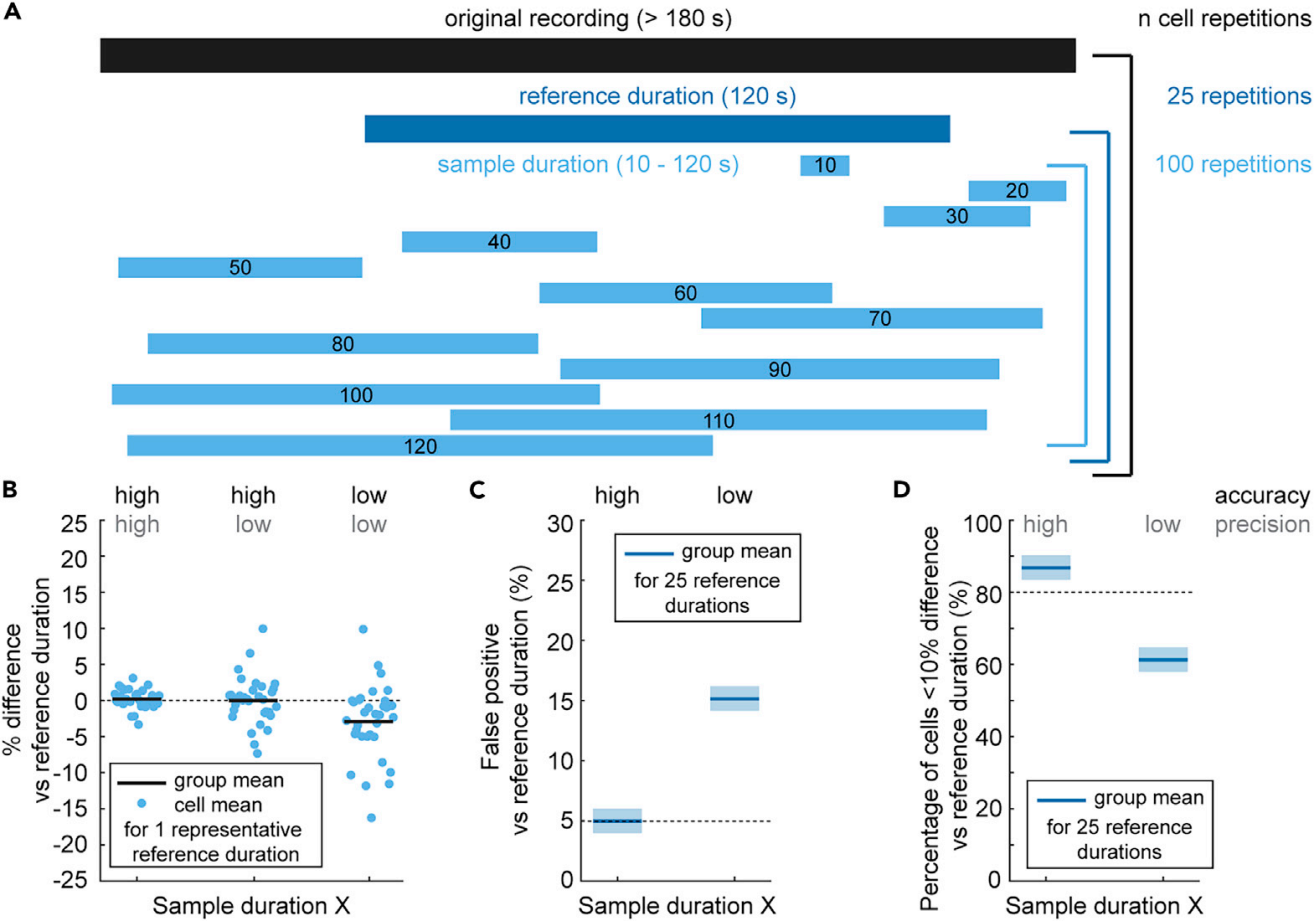
Methodological approach to investigate convergence of parameter estimates between shorter (sampling) and longer (reference) recording durations (A) We include recordings of nuclei neurons and Purkinje cells with an original recording duration of 180 s or longer (see Table 1 for mean recording durations). For each original recording duration, we take 25 reference durations of 120 s, randomly sampled across the full duration of the original recording. For each reference duration, we take 100 sample durations for each of the various lengths (10–120 s), randomly sampled across the full duration of the original recording. (B) We use one representative reference duration to show the mean difference in parameter estimate of 100 sample durations for each cell included in the analyses. This visualizes which sample durations provide an accurate and precise parameter estimate. (C) We perform paired t-tests for each reference duration and 100 paired sample durations and count the number of tests with p < 0.05. We repeat this for 25 reference durations and present the mean ± SEM This helps visualize which sample durations provide an accurate parameter estimate. (D) We count the percentage of cells with a parameter estimate in the sample duration that is within a 10% deviation of the reference duration. This visualizes which sample durations provide a precise parameter estimate.

In our first set of analyses, we investigated whether recording duration influenced the accuracy or precision of firing rate and pattern descriptors by comparing parameter estimates calculated from shorter sample and longer reference durations. We present images to help visualize the methodology for our quantitative analyses in Figure 1. We only included recordings that exhibited a stable action potential amplitude for a minimum of 180 s. We summarized the mean recording duration for all neurons included in this study in Table 1. For each round of analyses, we included one reference duration of 120 s with a start time randomly sampled from the original recording (Figure 1A). We then calculated the firing rate, CV, and CV2 based on the inter-spike intervals in this reference duration. We assumed that this long reference duration provided robust estimates of firing parameters, closest to the “true” values for the recorded neuron. We then compared the reference duration parameter estimates to those calculated based on the inter-spike intervals in shorter sample durations. The sample durations ranged from 10 to 120 s with a start time randomly sampled from the original recording (Figure 1A). For each round of analyses, we took 100 sample durations of varied lengths. We then repeated this sampling paradigm 25 times and compared the parameter estimates between sample and reference durations using three different analyses. Together, these analyses provided us with the sample duration at which the parameter estimates are accurate and precise, using the parameters estimates calculated in the reference recordings as the standard.

**Table 1:** Summary statistics for neurons included in our analyses and figures

Fig	Neuron Type	Spike Type	n	Awake/Anesthetized	Recording Length (s)	Firing rate (spike/s)	CV	CV2
1	Nuclei neuron	All spikes	33	Awake	224.5 ± 44.9	67.2 ± 24.1	0.49 ± 0.21	0.41 ± 0.11
2	Purkinje cell	Simple spikes	16	Awake	225.1 ± 39.1	66.6 ± 22.9	0.50 ± 0.09	0.43 ± 0.10
2	Purkinje cell	Complex spikes	16	Awake	225.1 ± 39.1	1.43 ± 0.39	0.74 ± 0.08	0.84 ± 0.07
3	Nuclei neuron	All spikes	17	Anesthetized	317.6 ± 63.5	34.4 ± 12.6 ***p<0.001***	0.38 ± 0.15 p = 0.051	0.39 ± 0.13 p = 0.586
3	Purkinje cell	Simple spikes	33	Anesthetized	304.0 ± 37.4	44.4 ± 14.4 ***p<0.001***	0.38 ± 0.10 p = 0.054	0.35 ± 0.10 ***p = 0.005***
3	Purkinje cell	Complex spikes	33	Anesthetized	304.0 ± 37.4	1.15 ± 0.43 ***p = 0.035***	0.75 ± 0.18 p = 0.675	0.90 ± 0.15 p = 0.143
4	Nuclei neuron	All spikes	16	Awake	220.7 ± 86.2	54.1 ± 31.0 p = 0.112	0.82 ± 0.27 ***p<0.001***	0.56 ± 0.18 ***p = 0.001***
4	Purkinje cell	Simple spikes	14	Awake	215.8 ± 53.3	32.4 ± 19.3 ***p<0.001***	1.45 ± 0.66 ***p<0.001***	0.72 ± 0.20 ***p<0.001***

Values represent mean ± SD. t-tests were used to find statistically significant differences in firing rate, CV, and CV2 of nuclei neurons and Purkinje cells recorded in awake control mice versus anesthetized control mice, or awake control mice versus awake experimental mice. p-values smaller than 0.05 are in bold italics.

First, we calculated the mean difference in parameter estimate between the 100 sample durations and one representative reference duration for all recordings included in the analyses. Accurate and precise sample duration parameter estimates clustered tightly around the reference duration parameter estimates, resulting in mean differences between sample and reference duration estimates close to zero (Figure 1B, left). Accurate but imprecise estimates centered around a mean difference of zero, but with larger deviations on either side (Figure 1B, middle). Inaccurate estimates clustered around a mean difference that deviates from zero (Figure 1B, right). This analysis determined the sample duration at which the parameter estimate population mean was the same when calculated in the sample and reference durations.

Second, we investigated whether systematically inaccurate parameter estimates in sample durations resulted in a statistically significant deviation from the “true” mean of the reference duration. This could result in a different conclusion about the parameter value in experimental settings. We used a paired t-test to investigate how many of the 100 sets of sample duration estimates were statistically different from the reference duration estimates. We repeated this for 25 reference durations and obtained the mean number of statistically significant comparisons (Figure 1C). In this analysis, any statistically significant result indicated systematic differences in the sample and reference duration parameter estimates. These differences could only be attributed to the total recording duration used for parameter extraction because parameter pairs were calculated from recordings from the same neuron in the same mouse during the same recording session. Nevertheless, an experimenter may have made a different conclusion about the population’s properties if the sample durations were analyzed instead of the reference durations. We referred to this as a “false positive” conclusion because a significant difference was observed where there should be none. Because we accepted statistical significance at p < 0.05, up to 5% of our comparisons might be statistically different (or false positive) based on chance. An accurate representation in the sample duration provided a false positive rate of 5% or below (Figure 1C, left), whereas a systematic inaccurate estimates resulted in false positive rates higher than 5% (Figure 1C, right). This analysis determined the sample duration at which the conclusion about parameter estimates was the same between sample and reference durations.

Third, we investigated the precision of parameter estimates. We tested this by investigating the percentage of cells with a 10% or smaller difference between sample and reference duration parameter estimate. We choose 10% as the deviation cutoff because the standard errors of the means are smaller than 10% of the population parameter estimates (Table 1). We defined that a sample duration provided precise parameter estimates when the difference between sample and reference duration parameter estimates was within 10% for 80% or more of the neurons included in the analyses. We repeated this comparison for 25 different reference durations paired with 100 sets of sample durations. A sample duration with precise parameter estimates had more than 80% of the parameter estimates within a 10% deviation of the reference duration estimate (Figure 1D, left), whereas imprecise sample duration estimates would have a low percentage within the 10% deviation of the reference duration estimates (Figure 1D, right). This analysis determined the sample duration at which the difference in sample and reference duration parameter estimates was minimal for most neurons in the population.

### Shorter sample durations underestimate the variability in cerebellar nuclei neurons

**Figure 2:**
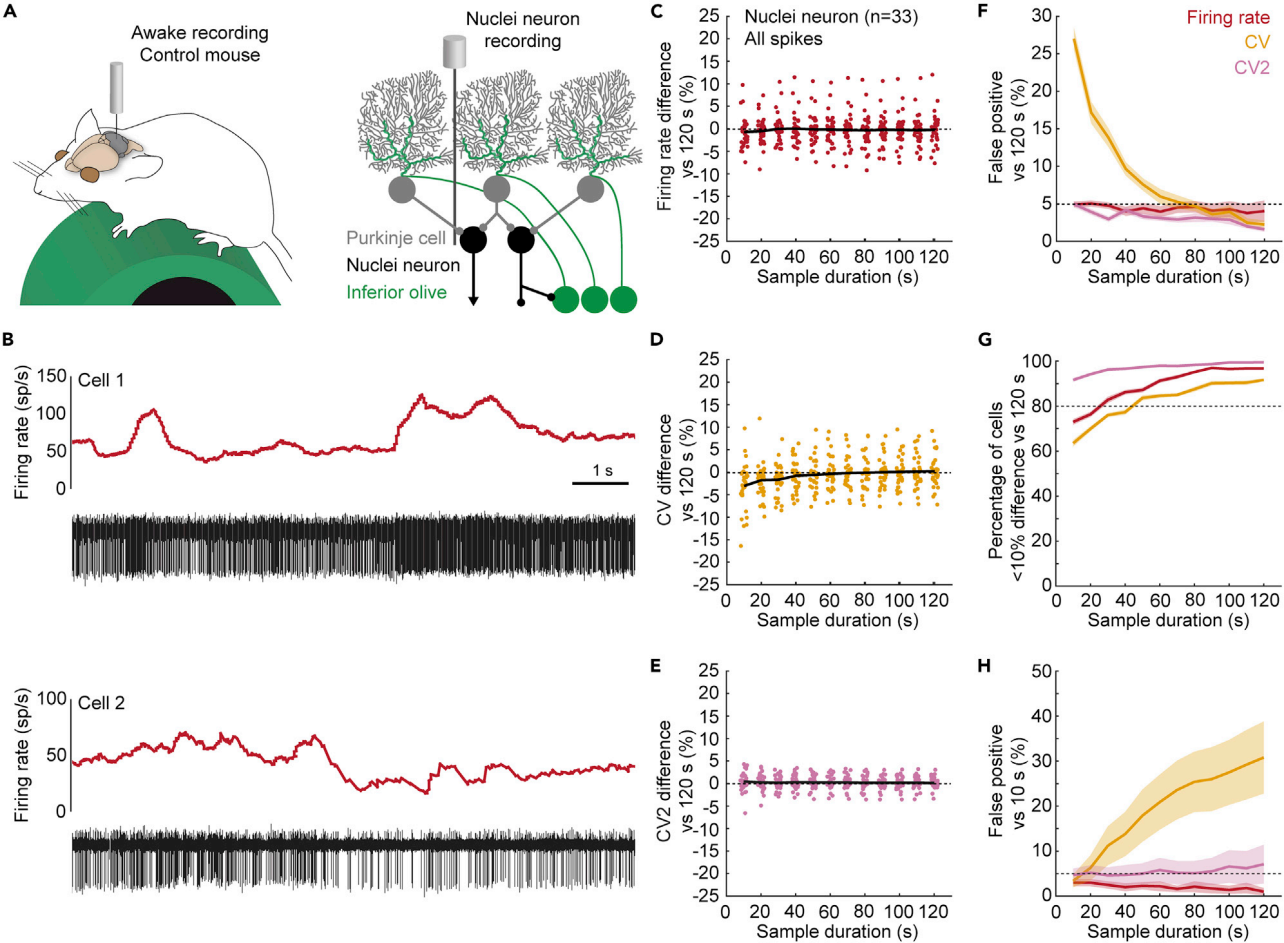
Variability in the firing properties of fast-firing neurons in awake mice (A) Schematic of recording setup for awake mice on a foam running wheel (top). For simplicity, in all schematics, we have not drawn the plates used for head-fixing the mouse or the recording chamber (see STAR Methods for details). Schematic of cerebellar circuit with nuclei neuron recording (bottom). (B) Two examples of nuclei neuron firing properties. Red traces represent the firing rate calculated over 0.5 s intervals. The black traces show raw electrophysiological recordings. Each vertical line is an extracellularly recorded action potential. (C–E) Mean difference in (C) firing rate, (D) CV, and (E) CV2 estimate between 100 sample durations and one representative reference duration. Each dot represents the average for 1 cell. Black line represents the mean for all cells included in the analyses. (F) Number of significant paired t-tests between parameter estimates in 120 s-long reference durations and 100 samples for each sample duration. (G) Percentage of parameter estimates in 100 samples for each sample duration within 10% deviation of the reference duration. (H) Number of significant paired t-tests between parameter estimates in 10 s-long reference durations and 100 samples for each sample duration. For F–H: Mean of 25 sets of 1 reference duration with 100 sample durations each = solid line; ± SEM = shaded region. Firing rate is shown in oxblood red, CV in orange, CV2 in pink.

We must ensure that the baseline firing properties are optimally represented in order to find differences in spiking activity between disease models or experimental groups. This means that baseline activity recordings must have a sample duration that encompasses a representative range of spiking activity during the recording session. Head-fixing a mouse over a rotating running wheel (Figure 2A, top) is a common arrangement for recording baseline spiking activity. We expect a large amount of sensorimotor modulation from this setup as the mouse can move all parts of its body, except for the head. Additionally, the mouse sits exposed to the recording room, allowing it to interact with and perceive a range of sights, smells, and sounds. Indeed, we found that the firing rate of cerebellar nuclei neurons fluctuated during our recording sessions (Figure 2B). These fluctuations could either be short bursts of higher firing rates (top) or prolonged periods of different firing rates (bottom). Fully capturing such fluctuations may require a longer sample duration.

We calculated the differences between parameter estimates in sample durations and reference durations to find the sample duration at which the mean difference converges to zero. We found that the differences between the sample and reference duration estimates occurred in either direction for firing rate (Figure 2C) or CV2 (Figure 2E), resulting in a mean difference of near zero for all sample durations. However, the CV estimate in sample durations between 10 and 50 s was lower than the CV estimate in the reference duration, which is represented as a negative mean difference in CV (Figure 2D).

We next investigated whether parameter estimates in sample durations significantly deviated from the “true” parameter estimate in the reference duration. The false positive rate was equal to, or smaller than, 5% for firing rate and CV2 estimates in all sample durations included in our analysis (Figure 2F). However, for the CV estimates, this false positive rate was higher than 5% for sample durations that were shorter than 60 s (Figure 2F). We further showed that the effect size converges toward a stable value in sample durations of 60 s or longer (Figure S1A). Together, these results confirmed that CV estimates in sample durations of 60 s or shorter were systematically lower than the CV estimates in the reference duration.

We also determined at which sample duration the parameter estimates were similar (or more precise) compared to parameter estimates in the reference duration. We found that firing rate estimates were precise in sample durations of 30 s or longer, CV estimates were precise in sample durations of 60 s or longer, and CV2 estimates were precise in sample durations of all lengths included in our analyses (Figure 2G).

We acknowledge that 60 s-long recordings might be a challenging goal for many experimental paradigms, especially when recording from awake and moving animals. Therefore, we also investigated how parameter estimates in our shortest sample duration (10 s) compared to parameter estimates in other recording durations. We found a high false positive rate when we compared CV estimates from a 10 s-long reference duration to 30 s or longer sample durations (Figures 2H and S1B). These findings showed that sample duration influences the estimation of parameter values that describe the global irregularity of spiking activity in nuclei neurons. Specifically, we found that CV was underestimated in shorter sample durations.

### Shorter sample durations underestimate the variability in Purkinje cell firing properties

**Figure 3:**
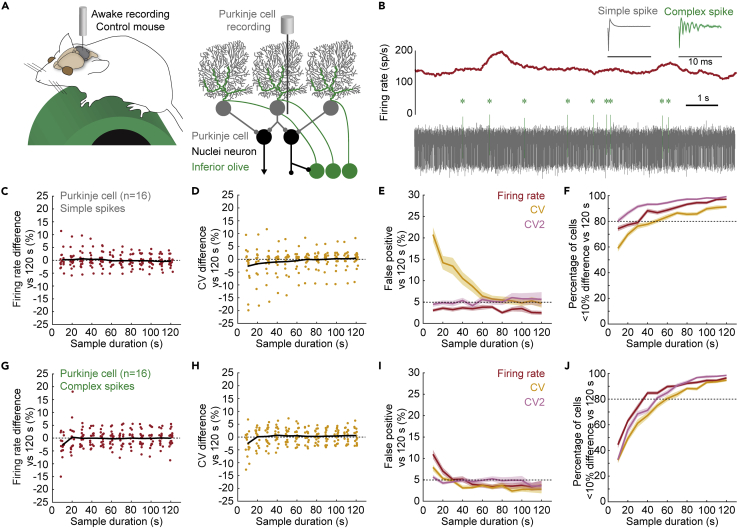
Variability in simple spike and complex spike firing properties of Purkinje cells in awake mice (A) Schematic of recording setup depicting an awake mouse on a wheel (left). Schematic of cerebellar circuit with Purkinje cell recording (right). (B) Example mean firing rate (calculated over 0.5 s) trace from a Purkinje cell recording with variability in firing pattern (middle, red). Bottom shows the underlying raw electrophysiological recording trace with simple spikes in gray and complex spikes in green. The higher power view traces (mean waveforms across a 20 s segment of a recording, top. 10 ms scale, below) demonstrate the unique spike profiles that distinguish simple spikes from complex spikes. (C and D) Mean difference in (C) firing rate, and (D) CV estimate for simple spikes between 100 sample durations and one representative reference duration. Each dot represents the average for 1 cell. Black line represents the mean for all cells included in the analyses. (E) Number of significant paired t-tests between parameter estimates in 120 s-long reference durations and 100 samples for each sample duration. (F) Percentage of parameter estimates in 100 samples for each sample duration within 10% deviation of reference duration. (G and H) as C and D for complex spikes. (I and J) as E and F for complex spikes. For E, F, I, and J: Mean of 25 sets of 1 reference duration with 100 sample durations each = solid line; ± SEM = shaded region. Firing rate is shown in oxblood red, CV in orange, CV2 in pink.

We next set out to investigate whether our observations in nuclei neurons are also true for a second principal neuron type in the cerebellum, Purkinje cells (Figure 3A). Purkinje cells fire simple spikes and complex spikes (Figure 3B), that differ in origin, shape, and firing rate.^76^^,^^77^ Simple spikes are spontaneously generated and occur between 40 and 200 spike/s (Table 1). Complex spikes are modulated by input from climbing fibers originating in the inferior olive, and occur between 1 and 2 spike/s (Table 1).^30^^,^^77^^,^^78^^,^^79^^,^^80^ Both simple spike and complex spike firing rates fluctuate during recording sessions in mice (Figure 3B). We analyzed how sample durations influence firing rate, CV, and CV2 estimates using the three analyses previously described (Table 1, n = 17).

Like the cerebellar nuclei neurons, Purkinje cell simple spike firing rate estimates in sample durations (10–120 s) deviated from those measured from a reference duration (120 s). However, the mean deviation converged to zero even for the shortest sample duration (10 s) (Figure 3C). In contrast to firing rate, CV was systematically underestimated in sample durations between 10 and 50 s (Figure 3D), which led to a high rate of false positive differences (Figures 3E and S1C). We found for sample durations longer than 50 s provided precise estimates for firing rate and CV (Figure 3F). This shows that short sample durations underestimate CV values and sample duration of ∼60 s were necessary for accurate and precise estimates of Purkinje cell simple spike firing rate and pattern.

Next, we investigated whether sample duration also influences the ability to estimate the firing properties for complex spikes, which are characterized by their lower firing rate. We found that, unlike nuclei neurons and Purkinje cell simple spikes, the complex spike firing rate was systematically underestimated in sample durations of 10 s (Figure 3G). In agreement with our findings in nuclei neurons and Purkinje cell simple spikes, we also found that complex spike CV was underestimated in sample durations of 10 s (Figure 3H). This resulted in a high rate of false positive differences between sample durations of 10–20 s and the reference durations for firing rate and CV estimates, but not CV2 estimates (Figures 3I and S1D). Finally, we found that sample durations of 70 s or longer provided precise estimates for firing rate, CV, and CV2 (Figure 3J). The sample duration necessary to provide precise estimates of firing pattern was longer for low-firing rate complex spikes than the high-firing rate simple spikes or nuclei neurons, showing that precise representation of low-firing rate complex spikes requires a longer sample duration.

### Firing rate variability estimates are less dependent on sample duration in anesthetized mice

**Figure 4:**
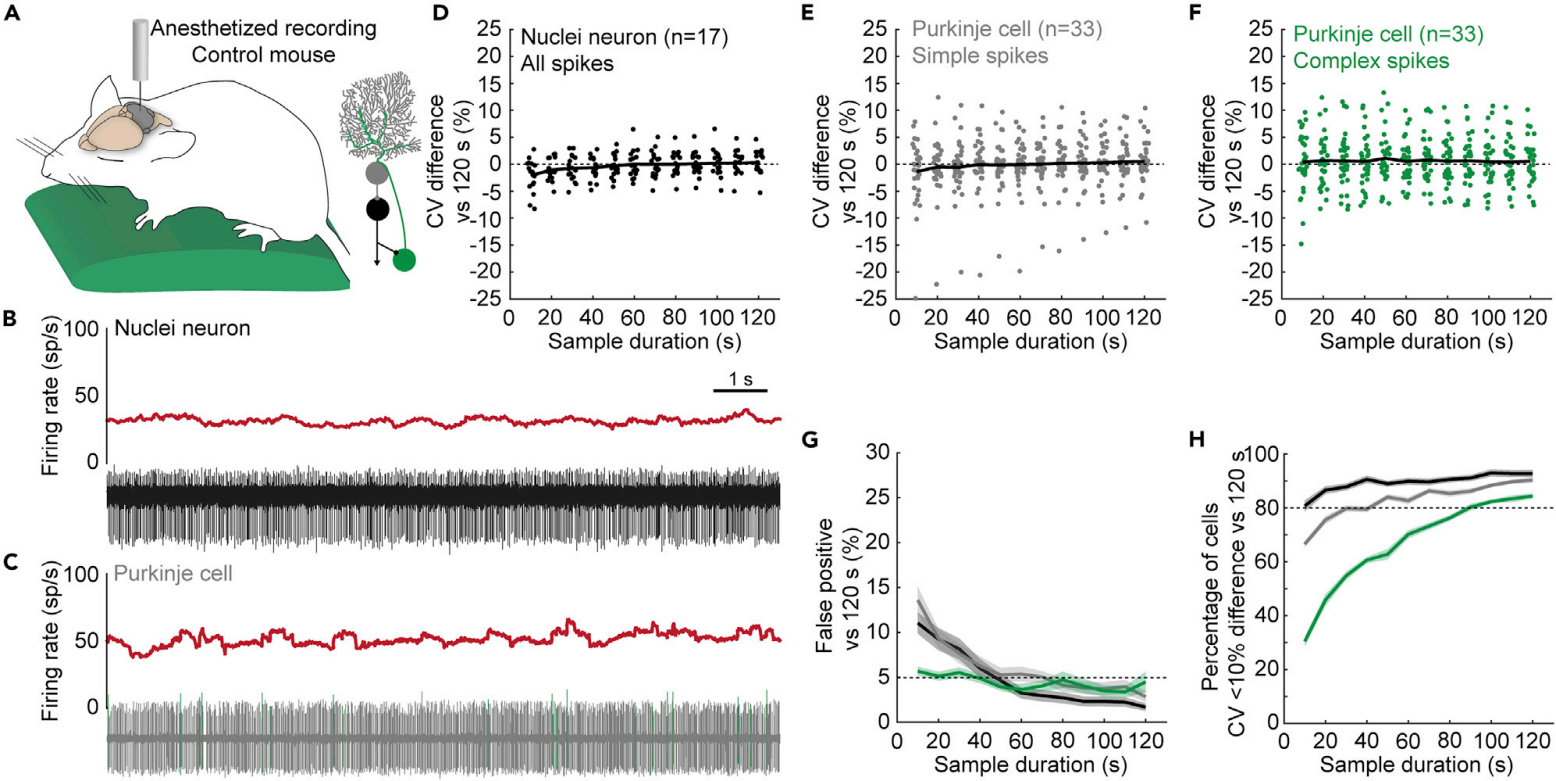
Variability in the firing properties of cerebellar nuclei neurons and Purkinje cells in anesthetized mice (A) Schematic of recording setup depicting an anesthetized mouse on a heating pad (left). Simplified schematic of cerebellar circuit (right). (B) Example trace of a nuclei neuron recording in an anesthetized mouse. Firing rate as calculated over 0.5 s intervals (top) and corresponding raw electrophysiological recording (bottom). (C) Example trace of a Purkinje cell recording from an anesthetized mouse. Firing rate as calculated over 0.5 s intervals (top) and corresponding raw electrophysiological recording (bottom). Simple spikes are depicted in gray and complex spikes in green. (D–F) Mean difference in (D) nuclei neuron spike, (E) Purkinje cell simple spike, and (F) Purkinje cell complex spike CV estimates between 100 sample durations and one representative reference duration. Each dot represents the average for 1 cell. Black line represents the mean for all cells included in the analyses. (G) Number of significant paired t-tests between CV estimates in 120 s-long reference durations and 100 samples for each sample duration. (H) Percentage of CV estimates in 100 samples for each sample duration within 10% deviation of the reference duration. For G and H: Mean of 25 sets of 1 reference duration with 100 sample durations each = solid line; ± SEM = shaded region. Nuclei neuron all spikes in green; Purkinje cell simple spikes in gray; Purkinje cell complex spikes in green.

We hypothesized that accurate descriptions of nuclei neuron and Purkinje cell baseline firing activity required longer sample durations because only longer durations captured sufficient firing rate modulating events.^29^^,^^30^^,^^56^^,^^67^^,^^81^ By this logic, firing rate variability was underestimated in shorter sample durations because the range of firing rates that modulate sensorimotor events was perhaps insufficiently sampled. If fewer different events modulate a neuron’s firing pattern, a shorter sample duration would be necessary to accurately describe that neuron’s firing pattern. Anesthesia induces immobility and prevents motor-event-related changes in Purkinje cell and nuclei neuron firing rate, while firing rates are still modulated by sensory stimuli (Figures 4A–4C).^65^^,^^82^^,^^83^^,^^84^ If adequate sampling of sensorimotor event was of primary importance for firing pattern representation, we would expect that accurate firing pattern estimates require a longer sample duration in awake mice than in anesthetized mice. We tested this by analyzing the difference between sample and reference duration parameter estimates in nuclei neuron and Purkinje cell recordings obtained in anesthetized mice.

We found that the difference in CV estimates between sample and reference durations converged to zero at sample durations of 40 s or longer for nuclei neurons in anesthetized mice (Table 1, n = 17) (Figure 4D). The percentage of false positives between the sample and reference durations also converged to 5% in sample durations over 40 s (Figures 4G and S1E). However, CV estimates were precise in all sample durations (Figure 4H).

We separately analyzed simple spikes and complex spikes from Purkinje cell recordings in anesthetized mice (Table 1, n = 33). For simple spikes, we found that the mean CV difference between sample and reference durations converged to zero at sample durations over 20 s (Figure 4E) and the percentage of false positive differences between sample and reference durations converged to 5% for sample durations over 40 s (Figures 4G and S1F). CV estimates were precise in sample durations of 30 s or longer (Figure 4H).

For complex spikes, we found that the mean difference in CV estimates between sample and reference durations was near zero for all sample durations (Figure 4F). We also found that the false positive rate was also under 5% for all sample durations (Figures 4G and S1G). Interestingly, CV estimates were only precise in sample durations over 80 s (Figure 4H). Thus, complex spike CV estimates in shorter sample durations were imprecise but did not skew systematically in either direction and therefore did not result in different population CV estimates between the shorter sample durations and the longer reference durations.

Taken together, we found that the sample duration necessary to accurately represent the CV in the cerebellar nuclei neurons and Purkinje cells recorded was shorter in anesthetized mice (∼40 s) than in awake mice (∼60 s). This shows that the sample duration necessary to optimally represent the neural variability likely relies on sufficient sampling of sensorimotor events that can modulate a neuron’s firing pattern. Even though we observed a trend to lower CV in neurons recorded in anesthetized mice compared to neurons recorded in awake mice (Table 1), none of the reductions were statistically significant (nuclei neuron: p = 0.051; Purkinje cell, simple spikes: p = 0.053; Purkinje cell, complex spikes: p = 0.675). This suggests that the observed differences in the recording length to optimally represent the neural variability are not solely due to a reduction in CV and may be secondary to a reduction in the sensorimotor events that induce temporary fluctuations in cerebellar firing rates in mice that are not awake or freely moving.

### Highly variable firing properties are accurately represented in short sample durations

**Figure 5:**
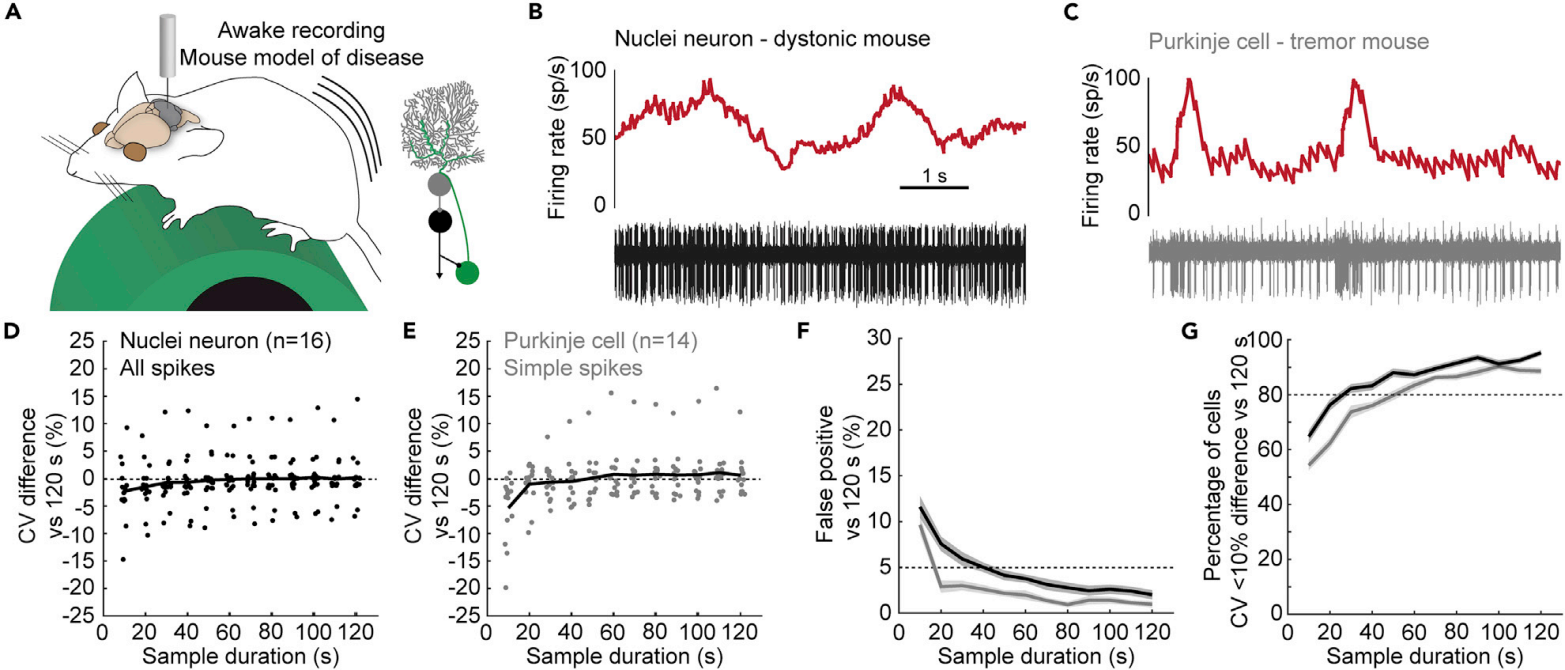
Variability in the firing properties of cerebellar nuclei neurons and Purkinje cells in mouse models of disease (A) Schematic of recording setup depicting an awake mouse on a foam running wheel (left). Simplified schematic of cerebellar circuit (right). Neurons included in the analysis for this figure come from *Ptf1a*^*Cre*^*;VGlut2*^*fl/fl*^ (dystonia-like) mice^8^; harmaline-injected (tremor) mice^7^; and *L7*^*Cre*^*;Vgat*^*fl/fl*^ (ataxia) mice.^7^ (B) Example trace of a nuclei neuron recording in an awake dystonic mouse. Firing rate as calculated over 0.5 s intervals (top) and matched raw electrophysiological recording (bottom). (C) Example trace of a Purkinje cell recording in an awake tremoring mouse. Firing rate as calculated over 0.5 s intervals (top) and matched raw electrophysiological recording (bottom). (D and E) Mean difference in (D) nuclei neuron spike, and (E) Purkinje cell simple spike CV estimates between 100 sample durations and one representative reference duration. Each dot represents the average for 1 cell. Black line represents the mean for all cells included in the analyses. (F) Number of significant paired t-tests between CV estimate in 120 s-long reference duration and 100 samples for each sample duration. (G) Percentage of CV estimates in 100 samples for each sample duration within 10% deviation of the reference duration. For F and G: Mean of 25 sets of 1 reference duration with 100 sample duration each = solid line; ± SEM = shaded region. Nuclei neuron all spikes in green; Purkinje cell simple spikes in gray.

Abnormal movements in mouse models for motor disorder can complicate the ability to obtain stable recordings. Our group and others have found high variability in cerebellar nuclei neuron and Purkinje cell firing rate in ataxia, dystonia, and tremor models (Figure 5A).^7^^,^^8^^,^^85^^,^^86^ Spike rate variability can thus arise from natural responses to sensorimotor events, but also from pathological changes in the firing properties of disease-associated neurons. We analyzed whether recording duration influenced the representation of variability in mouse models with unusually high CV. We included neurons from which we obtained a recording of 120 s or longer from mouse models of ataxia, dystonia, and tremor. Cerebellar nuclei neurons (Figure 5B) and Purkinje cells (Figure 5C) recorded in these disease mouse models had a higher CV value than neurons recorded in healthy control mice (Table 1) (nuclei neuron: p < 0.001; Purkinje cell, simple spike: p < 0.001).

In the cerebellar nuclei (Table 1, n = 16), we observed that the mean difference in CV estimates between sample durations and reference durations converges to zero (Figure 5D), the false positive rate converges to 5% (Figures 5F and S1H), and CV estimates were precise (Figure 5G) at a sample duration of 30 s or longer. This showed that the CV of nuclei neurons with highly variable firing patterns is accurately and precisely represented in recording durations of 30 s or longer.

For Purkinje cells with high CV (Table 1, n = 14), we only saw a systematic underestimation of CV in recordings with a sample duration of 10 s (Figure 5E) and we only observed false positive differences when comparing sample duration of 10 s to the reference duration (Figures 5F and S1I). CV estimates were precise in sample recordings of 50 s or longer (Figure 5G). Together, these data showed that, for Purkinje cells with high global irregularity, recording durations as short as 10 s provided an accurate, albeit imprecise, representation of the firing rate variability.

### Investigation of within and between mouse variability in parameter estimates

**Figure 6:**
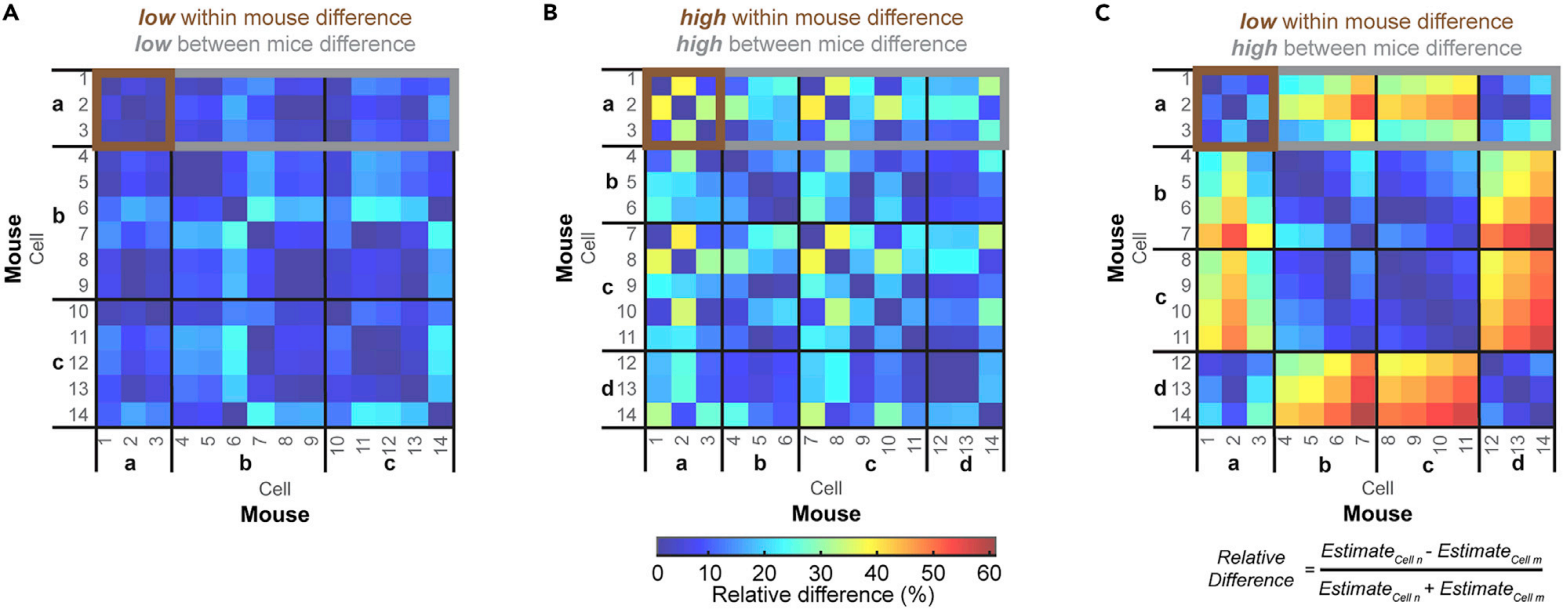
Difference in parameter estimates between cells recorded within the same mouse and between cells recorded from different mice (A) Representative comparison of the difference in parameter estimates in a population of cells with low within mouse variability and low between mice variability. (B) Representative comparison of the difference in parameter estimates in a population of cells with high within mouse variability and high between mice variability. (C) Representative comparison of the difference in parameter estimates in a population of cells with low within mouse variability and high between mice variability.

In addition to variability within the firing rate of single neurons, there is heterogeneity in firing properties across the population of recorded neurons. For example, differential responses to sensory information or motor commands, and therefore the mouse’s behavioral state during the recording, can cause heterogeneity in firing properties. As a result, multiple neural recordings from the same mouse may not be true independent samples, but rather nested data.^87^ We hypothesized that if recordings from the same mouse are truly nested data, and dependent on the behavioral state of the mouse, the relative difference in firing parameters would be smaller within the same mouse than between different mice. For our analysis, we only included neurons with a recording duration of 60 s or longer and only control mice from which we obtained three or more neurons that met this criterion. For each of the pairs of neurons, we calculated the relative difference in firing rate, CV, and CV2 as follows: absolute difference in parameter estimates between two neurons divided by the sum of the parameter estimates between two neurons (see STAR Methods) (Figure 6).

In Figure 6, we show a visual representation of the relative difference in parameter estimates between pairs of neurons using heatmaps, with blue representing the smallest difference and red representing the largest difference. In Figure 6A, we showed an example in which the difference in parameter estimates between neurons from same mice was low, and the difference in parameter estimates between neurons from different mice was also low. In Figure 6B, we showed an example in which the difference in parameter estimates between neurons from same mice was high, and the difference in parameter estimates between neurons from different mice was also high. In these two examples, the relative mean difference in parameter estimates within mice was the same as the relative mean difference in parameter estimates between mice. In Figure 6C, we showed an example in which the relative difference between neurons within each mouse was lower than the relative difference compared to neurons from different mice. In this example, sampling neurons from the one mouse may have caused a different conclusion about parameter values compared to sampling neurons from multiple mice.

### Within mouse variability does not drive systematic differences in nuclei neuron firing properties

**Figure 7:**
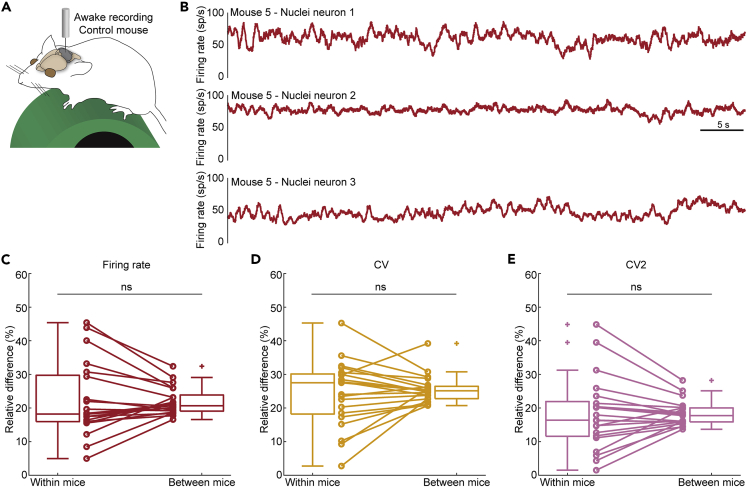
Differences in the firing properties of cerebellar nuclei neurons measured within and between awake mice (A) Schematic of recording setup depicting an awake mouse on a foam running wheel (left). Simplified schematic of cerebellar circuit (right). (B) Example mean firing rate traces of nuclei neuron recordings in awake control mice. All three traces come from the same mouse and represent the firing rate calculated over 0.5 s intervals. (C–E) Mean relative percent difference in (C) firing rate, (D) CV, and (E) CV2 between nuclei neurons within the same mice or between mice, statistically analyzed using a paired t-test. Not significant (ns).

When we investigated multiple nuclei neurons recorded from the same awake control mouse (Figure 7A), we saw differences in the firing rate and pattern (Figure 7B). We analyzed whether the variability in firing rate and pattern was independently sampled in neurons within the same mouse and between different mice by investigating the relative difference in firing rate, CV, and CV2 estimates in 92 neurons from 21 mice.

We found that the range of relative differences in firing rate, CV, and CV2 within mice was larger than the range of relative differences between mice, without a change in the mean difference in firing rate (p = 0.906), CV (p = 0.516), or CV2 (p = 0.941) (Figures 7C–7E and S2A–S2C). Thus, there was a relatively equal chance of sampling neurons with very different parameter values (high within mouse difference) as very similar parameter values (low within mouse difference). Additionally, the range of the parameter differences was smaller in the between mice comparisons because there are more neurons within the total population that have similar parameter estimates to each of the neurons included in the study. This showed that sampling multiple nuclei neurons within the same mouse did not influence the parameter estimates.

We also did not observe that the within mouse differences were smaller than the between mouse differences when we analyzed nuclei neurons recorded in anesthetized mice or mouse models for motor disease (Table 2). Together, these analyses showed that sampling of three or more cerebellar nuclei neurons from the same mouse during the same recording session did not bias the descriptions of cerebellar nuclei neuron firing rate and pattern.

**Table 2:** p-values for differences in firing properties in neurons recorded in experimental groups selected based on their distinct relevance to healthy or diseased circuits

Experimental group	Neuron type	Spike type	Mice (N), neurons (n)	p value Firing rate	p value CV	p value CV2
Anesthetized Control mice	Nuclei neuron	All spikes	N = 4, n = 19	0.696	0.235	0.283
Awake Harmaline-injected mice	Nuclei neuron	All spikes	N = 3, n = 11	0.312	0.245	0.741
Awake *Ptf1a*^*Cre*^*;VGlut2*^*fl/fl*^ mice	Nuclei neuron	All spikes	N = 4, n = 14	0.431	0.951	0.995
Awake *Pcp2*^*Cre*^*;Vgat*^*fl/fl*^ mice	Nuclei neuron	All spikes	N = 3, n = 9	0.301	0.982	0.572
Awake *Thap1*^*+/−*^ mice	Nuclei neuron	All spikes	N = 6, n = 29	0.749	0.839	0.790
Anesthetized Control mice	Purkinje cell	Simple spikes	N = 11, n = 46	0.445	0.452	0.197
Anesthetized Control mice	Purkinje cell	Complex spikes	N = 11, n = 46	***0.002***	***0.034***	0.247
Awake *Thap1*^*+/−*^ mice	Purkinje cell	Simple spikes	N = 4, n = 20	0.081	0.342	0.489
Awake *Thap1*^*+/−*^ mice	Purkinje cell	Complex spikes	N = 4, n = 20	0.345	0.574	0.905

Neurons included are from previously published studies.^7^^,^^13^^,^^38^^,^^75^ Significant differences are in bold italics.

### Variability between mice drives systematic differences in Purkinje cell firing rate

**Figure 8:**
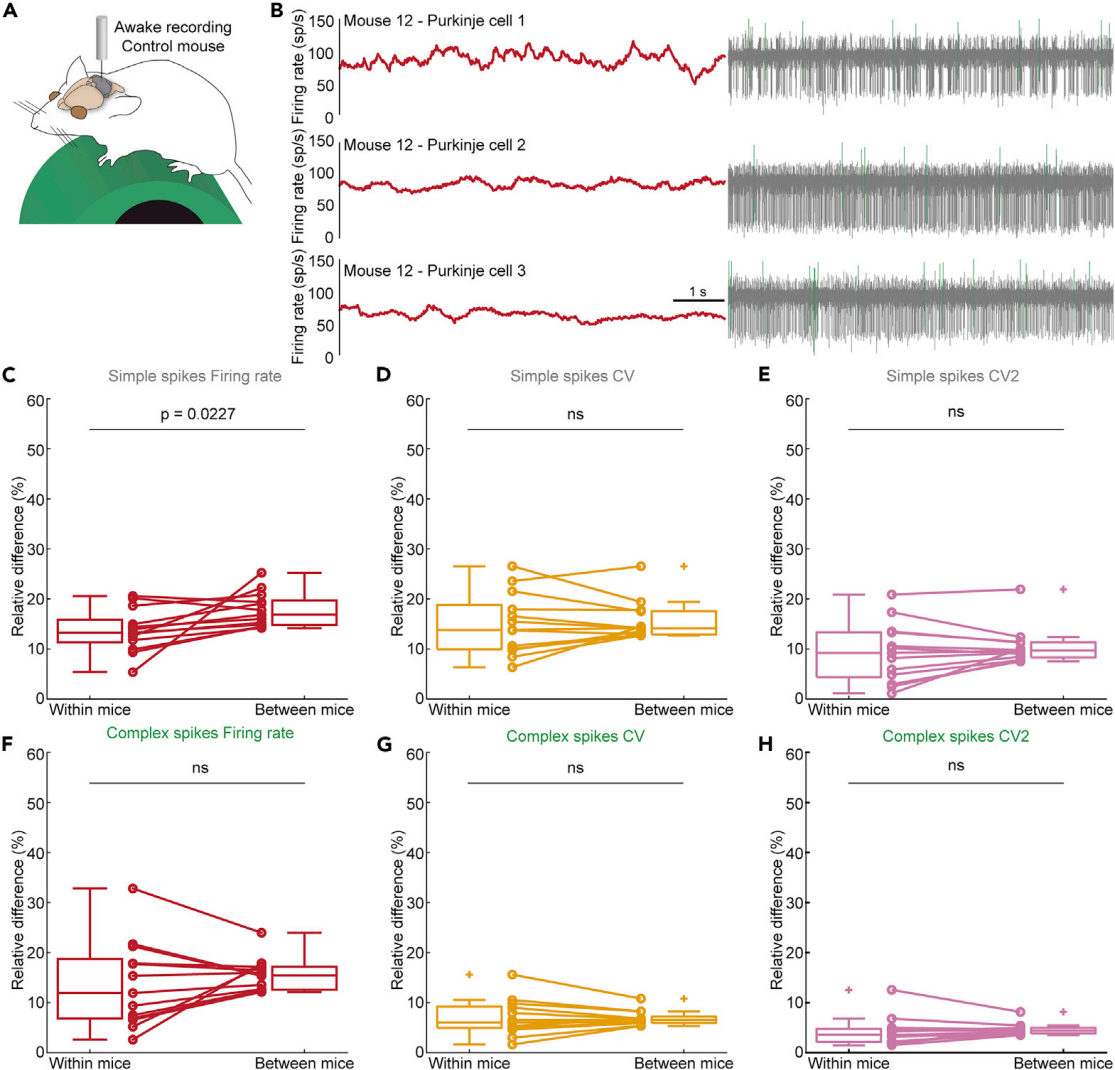
Differences in the firing properties of Purkinje cells measured within and between awake mice (A) Schematic of recording setup depicting an awake mouse on a foam running wheel (left). Simplified schematic of cerebellar circuit (right). Heatmap scale (bottom). (B) Example traces of Purkinje cell recordings in the same awake control mice. Mean firing rate calculated over 0.5 s intervals is represented in red (left). Simple spikes are represented in gray and complex spikes in green (right). (C–H) Mean relative percent difference in (C) simple spike firing rate, (D) simple spike CV, (E) simple spike CV2, (F) simple spike firing rate, (G) simple spike CV, and (H) simple spike CV2 between nuclei neurons within the same mice or between mice, statistically analyzed using a paired t-test. Not significant (ns).

We next set out to investigate whether the lack of mouse-dependent effects was also true for simple spikes and complex spikes recorded in Purkinje cells in awake mice (Figure 8A). Similar to nuclei neurons, we found that Purkinje cells recorded from the same mice during the same recording session had variable firing properties (Figure 8B). Next, we repeated the calculation of relative differences in firing rate, CV, and CV2 for simple spikes (Figures 8C–8E and S2D–S2F) and complex spikes (Figures 8F–8H and S2G–S2I). Again, we included only neurons from which we obtained a stable recording with a duration of 60 s or more, and only control mice with at least three separate neural recordings. We included a total of 56 Purkinje cell recordings from 13 awake, control mice in our analysis.

We found a statistically significant higher relative difference in simple spike firing rate between mice compared to within mice (p = 0.0227, Figure 8C). Specifically, in eleven out of thirteen mice, the relative difference in firing rates within mouse was smaller than the relative difference in firing rate between mice. We did not observe any statistically significant effects on the relative difference in complex spike firing rate between mice compared to within mice (p = 0.326). Similarly, we did not find mouse-specific effects on simple spikes (p = 0.560) and complex spikes (p = 0.811) CV, or on simple spikes (p = 0.250) and complex spikes (p = 0.473) CV2.

When we extended our analysis to Purkinje cells recorded in anesthetized mice, we found that complex firing rate (p = 0.002) and CV (p = 0.034) were more similar within mice than between mice (Table 2), without observing similar trends in simple spike firing rate or pattern. We also did not find mouse-specific effects in a mouse model for genetic dystonia (*Thap1*^*+/−*^ mice, Table 2). Thus, under certain conditions, Purkinje cell firing activity may be influenced by mouse-dependent effects and *in vivo* Purkinje cell recordings obtained from the same mouse cannot always be assumed to unequivocally represent independent measurements that properly report on the entire population.

## Discussion

Here, we explore the effects of data sampling strategies on common descriptors of neural activity. We find that the measurement of CV, describing global variability in firing rate, is biased toward lower values and susceptible to inaccuracy when recordings of *in vivo* awake basal neural activity are less than 60 s in duration. We observed that CV is underestimated at shorter sample durations for spiking activity with both high and low firing rate. We find that this duration threshold can be slightly decreased if sensorimotor stimuli are reduced or if CV is consistently elevated from control conditions. We also find that some cell populations may be less variable within mice than between mice, as demonstrated with Purkinje cells but not nuclei neurons.

### How might short sample durations result in skewed parameter estimates?

A previous study of *in vitro* electrophysiological recordings of pyramidal neurons in the sensorimotor cortex of rats found that a short observation length results in systematic underestimation of measures of local irregularity.^88^^,^^89^ In the analysis, Nawrot and colleagues argued that comparatively long inter-spike intervals, which are the primary driver of local irregularity, can become bisected by the boundaries of the recording window, resulting in a poor representation of these longer inter-spike intervals in the analysis, and thereby insufficiently sampling the inter-spike interval. Our finding that recording lengths of less than 20 s result in an underestimation of global irregularity is in line with the mathematical and empirical examples provided in these previous papers.^88^^,^^89^ We suggest that periods with relatively high variability too frequently fall outside the short recording duration window of analysis. The result would be analogous to the short recording length insufficiently sampling the inter-spike interval described by Nawrot and colleagues, but in our case would be insufficient sampling of variability. Similar logic may also explain our finding of a relative underestimation of firing rate from the low-firing rate complex spikes, wherein the recording window fails to include complex spike events that would result in a rate higher than the typical 1 spike/s because these events are relatively rare (Figures 3G and 3I). However, we find that using a recording duration of 20 s is long enough to reduce this bias.

We find that 60 s-long recordings are sufficient to avoid systematic underrepresentation of firing rate variability, but this duration may be longer, or shorter, for other brain regions, species, or experimental settings. Indeed, recording over a 10 to 300 s timescale, without a paired task, in a head-fixed mouse is just one of many potential experimental configurations. In systems neuroscience experiments, it is not uncommon to record freely moving animals for tens of minutes or longer. A freely moving animal would experience an even wider array of sensorimotor signals compared to our head-fixed animals. Based on the data we present here, we would expect that a recording of at least 60 s or longer would be required to elucidate basal firing patterns in this type of experiment. Regardless of the particular experimental setting or neuron type targeted for a given set of analyses, we recommend matching the sample durations for all analyses as we observed that the sample duration itself influences the estimate for firing pattern parameters.

### Where does the variability in firing patterns come from?

Neurons are subject to both intrinsic and extrinsic drivers of variability. For example, neurons throughout the brain—including cerebellar neurons—may toggle between up- and down-states which can also contribute to firing rate variability.^65^^,^^90^ As stated previously, recordings during *in vivo* baseline conditions include computations for ongoing natural tasks. Awake, head-fixed mice can perform many movements including walking on the wheel, and paw and whisker movement. These behaviors can directly modulate firing properties in cerebellar neurons.^28^^,^^29^^,^^91^^,^^92^ Similarly, the presentation of an abundance of olfactory, visual, auditory, and somatosensory stimuli during recordings in both awake and anesthetized mice results in fluctuations in the firing rate of cerebellar neurons.^30^^,^^83^^,^^84^ The same stimulus or action must be repeated many times to find the mean change in neural activity to the stimulus or action, or to map a change in the neural activity onto that stimulus or action. In transient recordings, it is unlikely that the same activity or stimulus is repeated sufficient times to relate changes of activity to a specific event. Time-synced video recordings of locomotor activity with neural activity are unlikely to provide insight into the source of variability in firing rate and pattern; video-taped motor events are unlikely captured sufficient times to align to specific modulation of neural activity. Additionally, video recordings also omit sensory events that can modulate cell and nuclei neuron activity. In task-independent, transient recordings, it is thus of importance to sufficiently sample diverse types of sensorimotor events that together determine the dynamic firing rates of a single neuron.

Our anesthetized condition findings lend evidence that sufficient sampling of the total range of sensorimotor modulations is required to prevent inaccurate estimates of global firing rate variability. Importantly, we show in these analyses that the number of spikes included in a recording duration does not necessarily drive the need for longer recording durations within the timescale we studied. All spike types studied here had a significantly lower baseline firing rate in the anesthetized state (Table 1). We would expect that the sample duration necessary to obtain accurate and precise parameter estimates would increase in the anesthetized condition if the number of spikes included in a recording was driving the ability to accurately describe firing properties. However, we find the opposite for all three spike types included in our analyses (Figure 4G). This suggests that adequate sensorimotor sampling is a stronger driver for the sample duration necessary to avoid inaccurate parameter estimates than the number of spike events sampled. It is also important to clarify that cells with lower firing rates may have a lower precision in parameter estimates, though without a change in accuracy, such as the complex spikes in the anesthetized state (Figures 4F and 4H).

The amount of sensorimotor information contained within the recording might play a similar role in the determination of necessary sample duration for mice with abnormally elevated CV (Figure 5). These mice had abnormal motor phenotypes and therefore the cerebellar neurons may not have carried as rich or as useful sensorimotor signals as in the control condition. Here, as in the anesthetized state, the sample duration at which parameter estimates were accurate was shorter than what was found in neurons recorded from the healthy awake mice. Therefore, the *in vivo* variability of firing rate in neurons in the cerebellum is likely the culmination of changes in firing rate due to movement, sensation, and intrinsic-cellular state. A recording duration commensurate with the range of sensorimotor signals that the neural activity is expected to carry in the experimental setting is necessary to accurately measure CV. When a longer duration is not possible, it is best to match recording durations so that a similar sample of neuromodulatory events may be captured in the recordings across neurons, mice, and experimental groups.

### Why is it important to measure both global and local firing rate variability?

We find that sample duration influences the ability to appreciate a neuron’s full repertoire of firing patterns. Specifically, shorter recordings resulted in a skew toward lower estimates of global variability (CV) in all neuron types, mouse models, and experimental paradigms included in our analysis. Meanwhile, our measurement of local variability, CV2, was more robust to duration differences. While both CV and CV2 describe the variability of spike activity, CV and CV2 are not interchangeable measures, and both are beneficial in describing the pattern of spike activity. Both CV and CV2 assist in distinguishing spike time irregularity in the form of variability (which results in high values for both CV and CV2) from irregularity in the form of rate fluctuation (which results in a high CV, but lower CV2 value).^74^ An example of irregularity in the form of large rate fluctuation with a much smaller concomitant increase in local variability has been observed in animal models with abnormal movements where bursts of very regular (low CV2) action potentials are interspersed by long pauses (resulting in high CV).^7^^,^^75^^,^^93^ Additionally, CV and CV2 can vary from control with both measures increasing together as the cell becomes more irregular,^8^ both decreasing as cell activity becomes more regular,^13^ both measures differing in opposite directions,^93^ or with one parameter changing while the other is stable.^7^^,^^94^ Therefore, each parameter provides an independent and unique measurement of the definition of firing rate variability. Excluding the global variability parameter for the sake of decreasing the necessary recording duration is ill advised without a comparable replacement.

### Will sample duration affect parameter estimates in task-specific paradigms?

It is also common to record spike activity in relation to specific behaviors or stimuli. Many studies have focused on understanding task-specific variability.^95^^,^^96^ Similar to the cerebellar nuclei, which are modulated by sensory and motor cues, firing rate variability is also found in motor and sensory cortices, even upon the presentation of the same movement or sensory cue.^97^^,^^98^^,^^99^^,^^100^ In this type of experiment, changes in spike parameters, most frequently firing rate, are measured relative to either an antecedent or other epoch of interest. Often, the epochs studied in these experiments are much shorter than what is described here, on the order of hundreds of milliseconds or less.^101^ Importantly, the goal of these experiments is often not to uncover the basal activity of a cell population, but rather to determine the response to sensory, motor, optogenetic, pharmacologic, or other events. Often, repeated observation of a cell’s response to the same event is used to determine the relative mean parameter values. Repeated observations of the same event are analogous to our finding that basal firing pattern should be measured over a longer duration because both allow averaging of the variability produced by variables such as movements, sensory inputs, and internal states. The ability of this experimental design to produce values that are both accurate and precise may depend on both the underlying firing rate and parameters measured, similar to what we have described here. Regardless of the experiment, we recommend matching the recording duration used to extract estimations of parameters describing firing properties for all neurons included in each experiment to avoid statistical significance arising from a difference in sample duration rather than the inherent firing properties in diverse populations of neurons.

### How might nested data influence firing rate and pattern estimates?

We also set out to investigate whether neural recordings obtained from the same animal are independent of each other or are susceptible to mouse-dependent variations. We found that for nuclei neurons in our database, there are no mouse-dependent effects on firing properties in awake or anesthetized control, or disease-model mice. In contrast, Purkinje cell simple spike firing rate may be more similar in neurons recorded from the same mouse than in neurons recorded from different mice. Finally, in the anesthetized state, we observe that Purkinje cell complex spike firing properties are more similar within mice than between mice, which may result from minor differences in anesthesia levels. Together, these results show that presentation and analyses of Purkinje cell firing properties may require accounting for mouse-specific effects. This could be accomplished by showing data in super plots^102^ as well as performing statistical analyses that take into account inter-mouse variability and correct for nested data.^87^^,^^103^ These results also underscore that data from multiple neurons recorded in multiple mice should be included when changes in Purkinje cell firing rates are anticipated and reported. Ideally, similar numbers of neurons should be included for each mouse used in the analyses.

At first glance, it may be surprising that there seem to be mouse-dependent variability in Purkinje cell firing properties, but not in nuclei neuron firing properties, as Purkinje cells provide direct inhibitory input onto nuclei neurons. However, anatomical and electrophysiological features may drive mouse effects only in Purkinje cells and not nuclei neurons. Different Purkinje cell types are organized in longitudinal zones whereas the cerebellar nuclei neuron subtypes are intermingled,^15^^,^^18^ even though they also respect the functional modules. The Purkinje cells within these zones have been found to have different baseline firing rates in a spatially organized manner.^1^^,^^40^^,^^49^ Thus, a single penetration of the electrode through the cerebellar cortex and nuclei is more likely to encounter the same molecular class of Purkinje cells with relatively similar firing rates, and more likely to encounter different molecularly defined classes of nuclei neurons with relatively varied firing rates. Therefore, when recording from neuron types or regions with known spatially organized diversity, the experimenter must either consistently sample neurons of the same molecular or regional identity or consistently sample across identities. Despite Purkinje cells directly innervating nuclei neurons, we did not find the same mouse-dependent variability in nuclei neuron activity. A contributing factor to this may be that the firing rate of nuclei neurons adapts relatively fast to changes in Purkinje cell firing rates.^104^ The lack of a direct inverse correlation between Purkinje cell firing rate and nuclei firing rate is further corroborated by our own findings in mice in which neurotransmitter release from Purkinje cells is blocked and we do not find a systematic increase in nuclei neuron firing rate.^7^^,^^38^ Thus, if mouse-dependent differences in Purkinje cell firing rates are due to differences in motor activity, sensory input, or arousal state during the recording session, nuclei neurons may adapt quickly and not show mouse-dependent effects.

### Will sampling methods influence data representation in non-cerebellar neurons?

One important feature of our study is that we performed our analyses on real neurons recorded *in vivo*, rather than *in silico* modeled spike trains. Our results are therefore independent of model assumptions and reflect robust biological ranges in firing properties. While prior theoretical papers have warned of the effects of nested data in electrophysiological recordings based on modeled assumptions of inter-animal effects,^103^ we show using experimentally obtained data that such effects can indeed occur *in vivo*. Recording duration (or number of spikes) is often selected as an inclusion criterion for analyses on firing properties, yet, to our knowledge, the influence of sample duration has not previously been reported by *in vivo* or *in silico* models of basal spike trains. Therefore, we believe that the effects of sample duration and between-mouse variability are robust experimental entities that have major relevance to a wide range of neural populations, as we observe that sample duration influences the estimation of firing rate variability in all spike types, neuron types, experimental settings, and mouse models included in our analyses.

### Conclusion

Taken together, while our analyses are focused on variability in firing properties in cerebellar neurons, they may generalize to the representation of firing properties in other neuron types and brain regions as well. We have detailed a set of analyses that provide a straightforward pipeline that scientists can adapt to investigate potential effects of recording duration or mouse-dependent effects on the reporting of firing properties of single neurons. For neural types in which little is known about how recording duration or inter-subject variability influences firing properties, the most conservative approach would be to match recording durations between all experimental groups, record from multiple individuals, include a similar number of recordings per individual, and perform statistical analyses that take inter-subject effects into account.

### Limitations of the study

Awake, head-fixed mice can perform a myriad of motor actions and sensory perceptions during a recording session. The collected data typically have neuronal activity variability with multiple possible sources that are often intentionally ignored to simplify analyses. For example, when comparing running vs non-running epochs, an experimenter may choose to ignore motor activities during non-running epochs such as whisking, postural adjustments, and grooming. One could also ignore variability during running epochs when the overall speed of the mouse or the speed of the limbs varies with the lengths of the step cycles. This study makes use of hundreds of recordings performed by multiple experimenters over several years to address this variability. Our approach presents its own limitations. To include the maximum number of mice and cells in this study, we could only account for the variability that was accounted for during the original experiments, groups of cells and mice based on the original study design, although common to our current study is the use of awake and anesthetized mice. However, the breadth of our database likely includes a comprehensive representation of the possible behaviors and sensations in both awake and anesthetized conditions. Therefore, while we confidently propose that our data are generalizable to the variability encountered in different motor or sensory states of the animal, future studies could test individual behaviors or sensory responses over extended periods of time. A recording approach that tracks specific dedicated behaviors would add to our understanding of the nuances of data sampling strategies suited for different motor and sensory states.

## STAR★Methods

### Key resources table

**Table:** 

REAGENT or RESOURCE	SOURCE	IDENTIFIER
**Chemicals, peptides, and recombinant proteins**		

Harmaline	Sigma Aldrich	#H1392

**Experimental models: Organisms/strains**		

*Ptf1a*^*Cre*^	JAX	RRID:IMSR_JAX:023329
*Vglut2*^*fl*^	JAX	RRID:IMSR_JAX:012898
*Pcp2*^*Cre*^	JAX	RRID:IMSR_JAX:010536
*Vgat*^*fl*^	JAX	RRID:IMSR_JAX:012897
*Thap1*^*+/−*^	Gift from Dr. Ehrlich	

**Software and algorithms**		

Software: Spike2	CED	RRID:SCR_000903
Software: MATLAB	MathWorks	RRID: SCR_001622

### Resource availability

#### Lead contact

Further information and requests for resources and reagents should be directed to and will be fulfilled by the lead contact, Dr. Roy V. Sillitoe (sillitoe@bcm.edu).

#### Materials availability

This study did not generate new unique reagents.

### Experimental model and subject details

All experiments described in this manuscript were reviewed and approved by the Institutional Animal Care and Use Committee (IACUC) at Baylor College of Medicine (BCM). Mice were housed in a Level 3, AALAS-certified facility on a 14-10 light-dark cycle. We used standard breeding paradigms to generate the different alleles, with the date a copulation plug was observed designated as embryonic day 0.5 and the date of birth as postnatal day 0. We used mice from both sexes and a range of ages (two to ten months old). Animals included in the analyses are control mice from mixed genetic backgrounds unless otherwise stated. The majority of neurons included in our analyses were reported in our previous publications (nuclei neurons and Purkinje cell recordings in awake mice^7^^,^^8^^,^^13^^,^^94^^,^^105^ and nuclei neurons and Purkinje cell recordings in anesthetized mice^38^^,^^75^). Procedures for electrophysiologically identifying different neurons, and collecting the data from different mice, cohorts and preparations by different experimenters was consistent between studies. The several previously reported experimental models are specified in the text. Mice indicated as “dystonic mice” are previously characterized *Ptf1a*^*Cre*^*;Vglut2*^*fl/fl*^ mice^8^ in which we genetically silence the climbing fiber to Purkinje cell synapse using *Ptf1a*^*Cre*^-mediated^106^ deletion of a *LoxP*-flanked sequence for the vesicular transporter for glutamate (*Vglut2*).^107^^,^^108^ Mice indicated as “ataxic mice” are previously characterized *Pcp2*^*Cre*^*;Vgat*^*fl/fl*^ mice^7^^,^^38^ in which we genetically silence Purkinje cells using *Pcp2*^*Cre*^-mediated^109^ deletion of a *LoxP*-flanked sequence for the vesicular transporter for GABA (*Vgat*).^110^ Tremor mice are previously characterized mice which received a 30 mg/kg intraperitoneal injection of harmaline (Sigma-Aldrich, #H1392).^7^ *Thap1*^*+/−*^ mice are a genetic model for heredity dystonia that display a mild pathophysiological tremor, which we characterized in a previous report.^13^ For our genetic mouse models, we collected ear-clippings at pre-weaning ages in order to extract DNA for PCR-genotyping. The PCR primers and PCR cycling conditions have all been reported in the publications describing the moude models. We used male and female mice for our studies in balanced numbers. All mice were between two and eight months old at time of recording.

### Method details

#### Surgery for recordings in awake mice

We performed a head-plate and craniotomy surgery to stabilize the head during awake recordings of cerebellar neurons.^6^^,^^7^^,^^8^^,^^64^ Throughout the surgery we kept the mice on a heated surgery pad and stabilized the head using ear bars in a stereotaxic surgery rig (David Kopf Instruments). We provided preemptive analgesics (slow-release buprenorphine at 1 mg/kg subcutaneous or buprenorphine at 0.6 mg/kg as well as slow-release meloxicam at 4 mg/kg or meloxicam at 5 mg/kg subcutaneous) and maintained mice under continuous anesthesia using nasally delivered isoflurane gas (2–3%). We removed fur from the surgery site and made an incision in the skin over the anterior part of the head. Next, we mounted a custom head-plate over bregma using C and B Metabond Adhesive Luting Cement (Parkell) and placed vertical visible wiring to annotate bregma during recordings. We then used a dental drill to make a circular craniotomy with a diameter of approximately 2 mm over the cerebellum (6.4 mm anterior and 1.3 lateral from Bregma) and placed a custom 3D-printed chamber over the craniotomy that we filled with antibiotic ointment prior to closing. We firmly attached the 3D-printed chamber and headplate to the mouse skull using Metabond and dental cement (dental cement powder #525000; solution #526000; A-M Systems). After surgery, the mice recovered from anesthesia in a clean cage resting on a heating platform. We monitored mice for stress and pain for a minimum of three days after surgery and throughout the experimental period. We provided mice with additional meloxicam and buprenorphine for three days after surgery.

#### Surgery for recordings in anesthetized mice

We performed a craniotomy surgery over the cerebellum to access cerebellar neurons during anesthetized recordings.^5^^,^^8^ Throughout the surgery and recording, we kept the mice on a heated surgery pad and stabilized the head using ear bars in a stereotaxic surgery rig (David Kopf Instruments). Prior to surgery, we anesthetized mice using a mixture of intraperitoneal administered ketamine and dexmedetomidine and nasally delivered isoflurane gas (1–2%). Next, we removed fur from the head and made an incision in the skin over the anterior part of the head. We used a dental drill to make a craniotomy with a diameter of approximately 5 mm over the cerebellum (6.4 mm anterior and 1.3 lateral from Bregma). Penetrations of the cerebellum proceeded as to capture the activity of as many Purkinje cells and cerebellar nuclei neurons as possible per recording session, which typically lasted for approximately 2–3 hours.

#### *In vivo* electrophysiological recordings

We stabilized the mouse’s head using the surgically attached head-plate (awake mice) or ear bars (anesthetized mice) and kept the mouse on a freely rotating foam wheel (awake mice) or heating pad (anesthetized mice) for the durations of the recording session. We used tungsten electrodes with an impedance of ∼8 MΩ for our recordings. The movement of the electrodes was controlled using a motorized micromanipulator (MP-225; Sutter Instrument Co). The electrical signals obtained by the electrodes were amplified and bandpass filtered (0.3–13 Hz) (ELC-03XS amplifier, NPI Electronic Instruments) before being digitized (CED Power 1401, CED). All *in vivo* electrophysiology signals were recorded and analyzed using Spike2 software (CED).

### Quantification and statistical analysis

#### Analysis of *in vivo* electrophysiological recordings

All single neuron recordings were quality controlled with signal to noise ratio as a primary consideration and spike sorted in Spike2. We also considered cell-specific features. We accepted all neurons that had clearly distinguishable complex spikes (a single large spike followed by three to five smaller amplitude spikelets, with a pause in simple spike activity last ∼20–50 ms following this whole signature), usually obtained between 0.5 and 2.5 mm from the brain surface as Purkinje cells. We identified the cerebellar nuclei neurons based on the depth at which they were recorded (between 2.5 and 3.5 mm from the brain surface). We only committed to analyzing the duration of recordings that maintained a stable action potential amplitude.

#### Presentation of *in vivo* electrophysiological recordings in figures

All traces included in the figures represent continuous raw electrophysiological recordings. Recording duration is represented in each figure panel with a time scale. Complex spikes are pseudo colored in Illustrator based on spike sorting in Spike2. We calculated the firing rate at each spike in the visualized spike trains (Figures 2B, 3B, 4B, 4C, 5B, 5C, 7B and 8B) by calculating the firing rate (spikes/s) in the previous 0.5 seconds.

#### Calculation of parameters describing neural firing properties

We described the firing properties of single Purkinje and cerebellar nuclei neuron recordings using a combination of three parameters that are most often reported when describing the firing properties of cerebellar neurons.^2^^,^^13^^,^^74^ First, we calculated the firing rate as follows: $firing\;rate=\frac{number\;of\;spikes}{recording\;duration}$. Second, we measured the global irregularity in firing rate, CV, based on the interspike interval (ISI) between spikes: $CV=\frac{stdev\left(ISI\right)}{mean\left(ISI\right)}$. The CV is a standardized measure for the global irregularity in the firing rate as it is calculated based on the mean deviation of ISI from the mean ISI and normalized to the mean ISI. As a result, a neuron with a highly fluctuating firing rate, will have a high CV, whereas a tonically firing neuron will have a low CV. Third, we calculated the CV2 for all pairs of subsequent spikes pairs (pair 1: n = 1, n = 2; pair 2: n = 2, n = 3; etc.) using the following formula: $CV2=\;mean\frac{2\left|ISI_{n}-\;ISI_{n-1}\right|}{ISI_{n}+\;ISI_{n-1}}$. This measure indicates the local irregularity in the firing rate of a neuron as it takes in account the mean difference in ISI between each adjacent pair of spikes in the recording.^74^ All analyses were performed using custom-written code in MATLAB (The MathWorks Inc., version 2021a).

#### Quantification of the influence of recording length on firing pattern parameters

We determined whether recording length biases the description of firing properties in single neuron recordings by calculating how the firing rate, CV, and CV2 differed when calculated over shorter versus longer periods taken from the same recording. In this calculation, we only included neurons from which we had a high-quality, continuous recording of three minutes minimum (180 s) (Figure 1). We then randomly sampled a continuous 120 s period from this longer recording and calculated the firing rate, CV, and CV2 in this period as an estimation of the true firing properties during our recording session. We call this the reference duration. We choose to calculate the firing properties based on a randomly sampled 120 s long recording time window instead of using the complete recording length to equalize the reference duration across neurons. This approach also minimized the potential confound of electrode movement that is most likely to occur at the start or end of each recording. An additional benefit of this approach is that the parameter estimation from each sample duration is not always performed on a set of spike time intervals that is fully included in the parameter estimates for reference duration. In the example in Figure 1A, sample durations 20 and 50 s have no temporal overlap with the reference duration, and sample durations 30, 70, 80, 90, 100, 110, and 120 s have only partial temporal overlap with the reference duration.

We next calculated the difference between the parameter estimates of our 120 s reference durations and those calculated over sample durations of 10 s to 120 s (10 s, 20 s, 30 s, … 120 s). These sample durations were also randomly sampled from the entire duration of the recording we had available for each neuron. We took each sample durations at least 100 times and calculated the difference as follows: $difference=\frac{Estimate_{sample}-Estimate_{reference}}{Estimate_{reference}}\times 100\%$. The mean difference between 100 sample durations and one reference duration is represented in the figures that show the differences in parameter value (Figure 1B).

We then performed a paired t-test between the parameter as calculated based on the sample durations and a reference duration (*t =* 120 s or *t =* 10 s) and counted the number of tests with a p-value lower than 0.05 (Figure 1C). We used a Lilliefors test to confirm that the parameters we have chosen for analyses (firing rate, CV, and CV2) follow a normal distribution. We also calculated the Cohen’s d effect size for all paired t-tests: $effect\;size=\frac{mean\;\left|\left(Estimate_{samplee}-Estimate_{reference}\right)\right|\;}{stdev\;\left|\left(Estimate_{sample}-Estimate_{reference}\right)\right|}$. We presented the effect sizes in Figure S1. Finally, we counted the proportion of neurons with parameter calculations from the sample duration that were within 10% of the parameter calculation from the reference duration (Figure 1D). For our analyses of percentage of false positive differences, effect size calculation, and analyses of proportion of neurons with parameter estimates within 10% of the reference duration estimates, we repeated our analysis 25 times. Each repetition included 1 new reference duration and 100 sample durations of each length. For these three calculations, the data shown in the figures represent the mean ± the standard error from the mean across 25 reference durations.

#### Quantification of the inter- versus intra-mouse variability in neural firing properties

For our analyses that examine the of effect within versus between mouse variability, we only included neurons for which we obtained an original recording length of 60 s or longer. We also only included mice for which we obtained at least three neurons that met this criterion. We determined the relative difference in parameter values in neurons as follows: $difference=\frac{\left|Estimate_{Cell\;n}-Estimate_{Cell\;m}\right|}{Estimate_{Cell\;n}+\;Estimate_{Cell\;m}}\times 100\%$. Here, *Estimate* is the calculated parameter (firing rate, CV, or CV2) calculated for each neuron *n* and *m*. We then calculated the mean relative difference for all neuron pairs where neuron *n* and *m* were obtained from the same mouse (but not the same neuron). We designated this as the relative difference within mice, for each mouse. We also calculated the mean relative difference for neuron pairs where neuron *n* and *m* were not obtained from the same animal. We designated this as the relative difference between mice, for each mouse. We investigated whether the deviation between the relative difference within and between mice was statistically significant using a paired t-test.

## Data Availability

All data produced in this study are included in the published article and its supplementary information, or are available from the lead contact upon request. This paper does not report original code. Any additional information required to reanalyze the data reported in this paper is available from the lead contact upon request.
