# Supplementary material for: Influence of data sampling methods on the representation of neural spiking activity in vivo
Source: iScience. 2022 Oct 22;25(11):105429. doi: 10.1016/j.isci.2022.105429 (PMC9641233; doi:10.1016/j.isci.2022.105429)

iScience, Volume *25*

Supplemental information

Influence of data sampling methods on the representation of neural spiking activity *in vivo*

Meike E. van der Heijden, Amanda M. Brown, and Roy V. Sillitoe

Figure S1. Cohen's *d* effect size for comparison of 100 sample durations to twenty-five representative reference durations. Figures represent the mean ± SEM. Related to Figures 2-5.

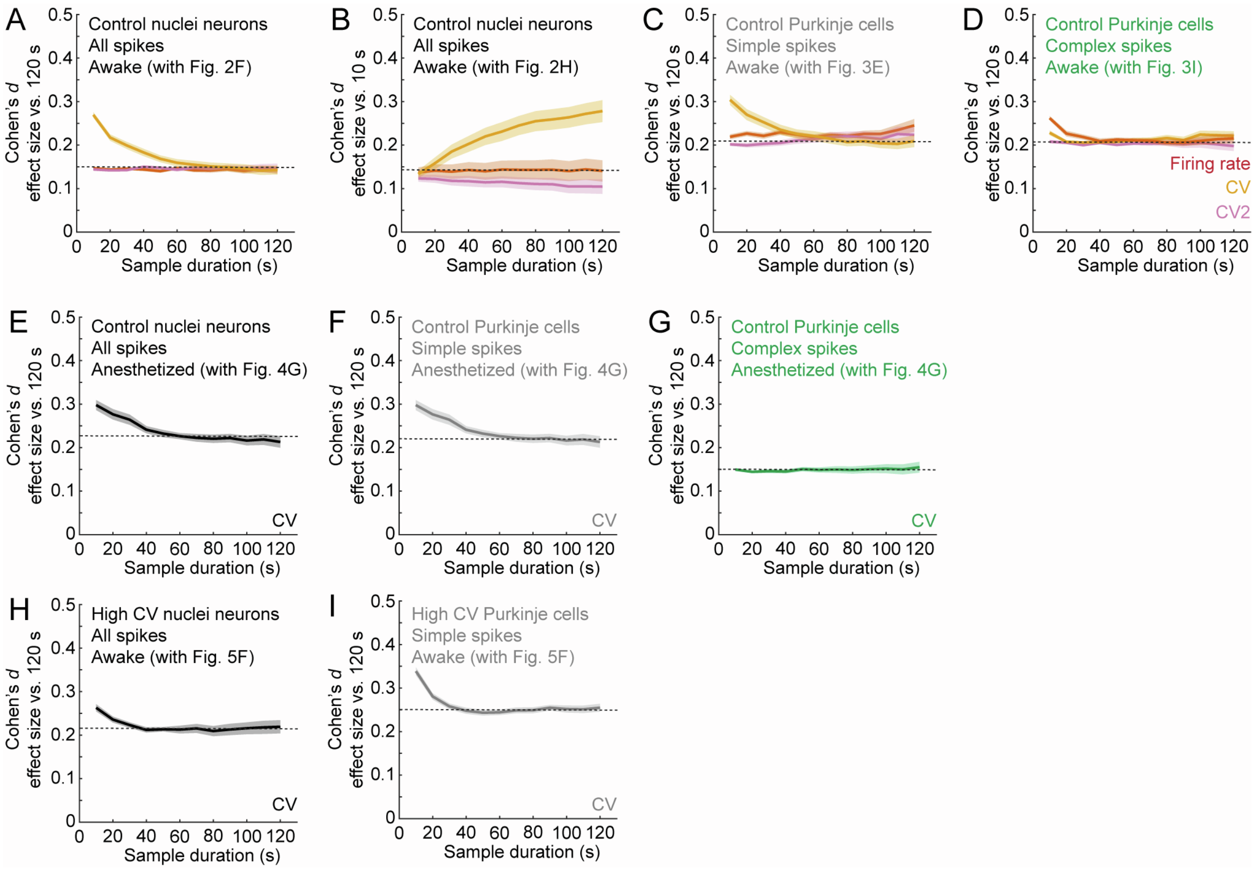

Figure S2. Relative difference in **A.** firing rate, **B.** CV, and **C.** CV2 between all nuclei neuron pairs in 21 mice. Relative difference in **D.** simple spike firing rate, **E.** simple spike CV, **F.** simple spike CV2, **G.** complex spike firing rate, **H.** complex spike CV, **I.** complex spike CV2, between all Purkinje cell pairs in 13 mice. Each row and column represent 1 cell. Thin white lines segregate cells from different mice. Related to Figures 7-8.

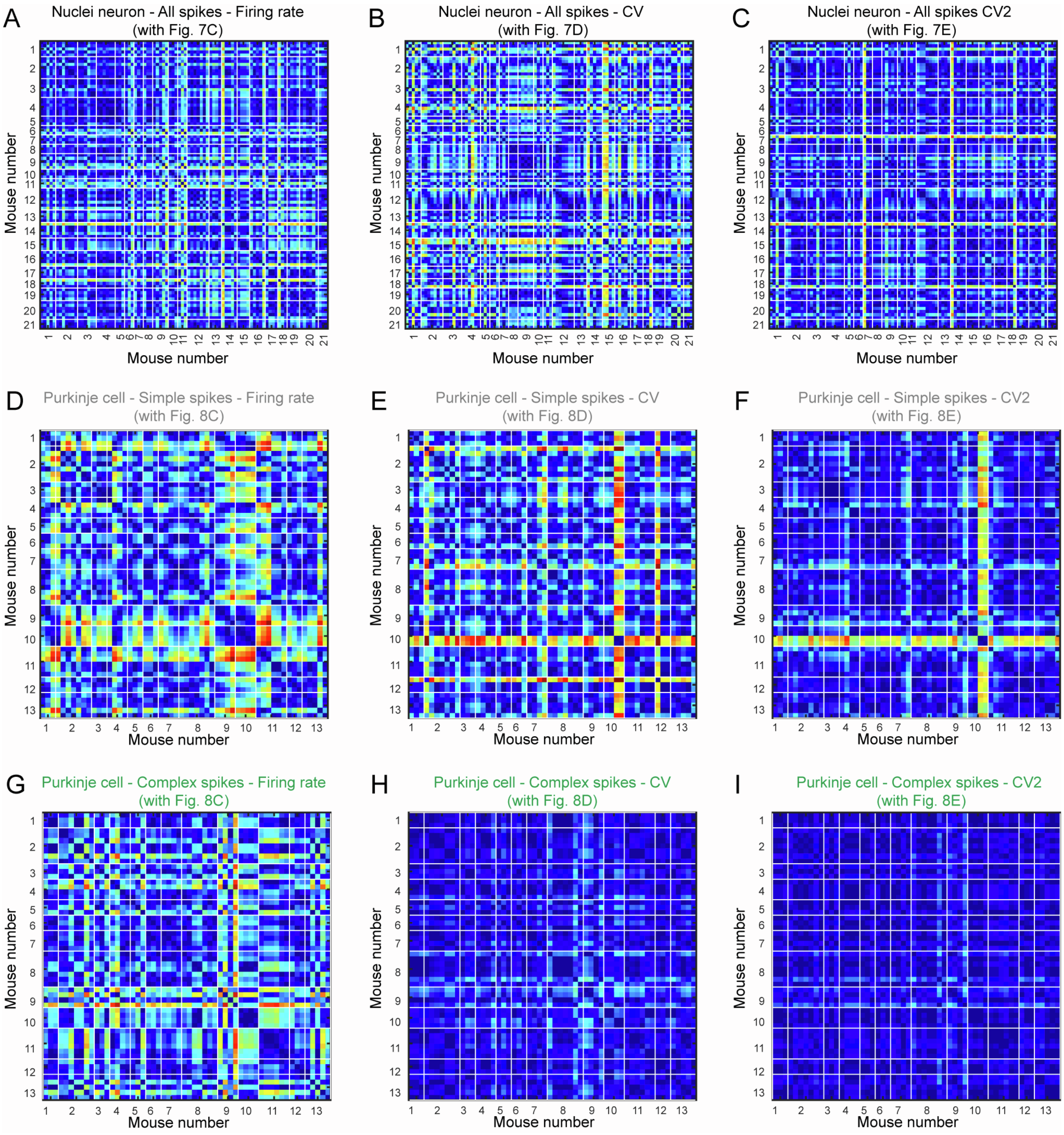

Supplement: Document S1. Figures S1 and S2 [file mmc1.pdf]
